# Editorial: New Technologies for Women’s Health

**DOI:** 10.3389/fbioe.2022.969389

**Published:** 2022-07-13

**Authors:** Lana McClements, Dunja Aksentijevic, Vesna Garovic

**Affiliations:** ^1^ School of Life Sciences, Faculty of Science, University of Technology Sydney, Sydney, NSW, Australia; ^2^ Centre for Biochemical Pharmacology, William Harvey Research Institute, Barts and the London School of Medicine and Dentistry, Queen Mary University of London, London, United Kingdom; ^3^ Division of Nephrology and Hypertension, Department of Internal Medicine, Mayo Clinic College of Medicine, Rochester, MN, United States; ^4^ Division of Nephrology and Hypertension, Department of Obstetrics and Gynaecology, Mayo Clinic College of Medicine, Rochester, MN, United States

**Keywords:** women’s health, preeclampsia, ovarian cancer, pregnancy, 3D bio printing, biomarkers, miRNA, placenta

## Introduction

Over the last decade, sex differences have been widely explored and have demonstrated the importance of understanding women’s and men’s health in the context of population health as a whole. These differences start *in-utero*, where male foetuses are larger by the second semester, whilst maternal immune response is stronger towards a male foetus creating a more pronounced pro-inflammatory environment compared to a female foetus. Male foetuses are also at higher risk of infection that can lead to premature birth (Inkster et al., 2021). In this Research Topic “*New Technologies for Women’s Health*,” we focused on emerging technologies and discoveries specific to women’s health that includes eleven articles divided into four themes: ovarian cancer (2), pregnancy (4), preeclampsia (3) and digital technologies (2).

### Ovarian Cancer (2)

Two articles have been published related to ovarian cancer research. One describes innovative three D (3D) cellular technologies for modelling of ovarian cancer, which can be used to accelerate discovery towards implementation of personalized medicine Yee et al. Ovarian cancer is a heterogenous disease with different subtypes, some of them, such as high grade serous ovarian cancer, poorly responding to currently available therapies. 3D modelling systems of ovarian cancer can recapitulate cell-cell and cell-microenvironment interactions better than 2D, hence improving chances of successful translation of emerging treatments and improving responses of particular ovarian cancer subtypes to available therapies. The second article Hossain et al. outlines novel approaches to biomarker discovery for early diagnosis of ovarian cancer, which is an area of unmet clinical need. Potential candidates were discussed including circulating miRNA and cell-free DNA (cf-DNA), exosomes, and Chloride Intracellular Ion Channels (CLIC) family of proteins.

### Pregnancy-Related Research and Technologies (4)

This sub-theme covers a wide range of research during pregnancy. For example, Bertini et al. is a systematic review of machine learning approaches for prediction of complications in pregnancy. The data collection methods included electronic medical records, medical images, biological markers, and to a lesser extent, sensors and foetal heart rate. They concluded that the machine learning approach was the most effective when using medical images for prediction of preterm birth and neonatal mortality.

Ghamrawi et al. compared methylation status in pregnancy using buffy coat DNA vs. DNA isolated from polymorphonuclear (PMN) and lymphocytic fractions obtained within 24 h prior to delivery from 29 normotensive pregnant women. Their study determined that buffy coat DNA rather than lymphocytic DNA is more representative of methylation patterns in white blood cells during normal pregnancy.

A retrospective cohort study reported on the effectiveness of fetoscopic laser coagulation in ameliorating the metabolomic alteration caused by twin-twin transfusion syndrome in placental tissue and cord plasma Liu et al. They also state that these altered metabolites are involved mainly in fatty acid and lipid-like molecule metabolism and that certain lipids and lipid-like molecules are correlated with neonatal birth weight or ejection fraction.

Novel therapeutic strategies involving an anti-oxidant and anti-inflammatory agent, dendrimer-based N-acetyl cysteine (DNAC), were evaluated in Liu et al. in terms of its ability to ameliorate placental inflammation, as a key cause of preterm birth and post-pregnancy adverse health consequences. The results of this *in vivo* study demonstrated that DNAC reduced the M1 pro-inflammatory macrophage cell subpopulation whilst increasing M2 anti-inflammatory macrophages, hence improving the placental immune profile in the intrauterine inflammation model.

### Preeclampsia (3)

Preeclampsia is the leading cause of mortality and morbidity in pregnancy, yet currently this pregnancy-specific hypertensive disorder does not have a cure (Thornton et al., 2013). Better understanding of the pathogenic mechanisms leading to preeclampsia, particularly those affecting placental health, is key to developing better management strategies for preeclampsia. In this meta-analysis, Cirkovic et al. using publically available data, a number of placental miRNAs were identified as having the most promising role in the pathogenesis of preeclampsia. These included placental miRNA-16, miRNA-20b, miRNA-23a, miRNA-29b, miRNA-155 and miRNA-210, miRNA-376c, and peripheral blood miRNA-155 and miRNA-16; the vast majority of these were increased except for miRNA-376c and miRNA-16, which were found to be decreased in preeclampsia.

On the other hand, another study Freimane et al. performed a systematic review that identified the most promising biomarker candidates for early pregnancy prediction of preeclampsia risk in women with type 1 diabetes mellitus (T1DM). Pregnant women with T1DM have a four-fold increased risk of developing preeclampsia (Weissgerber and Mudd, 2015). This important review discussed 32 biomarkers, suggesting that first trimester HbA1c, urinary albumin, neutrophil gelatinase-associated lipocalin and adipokines likely have the best potential for prediction of preeclampsia in women with T1DM. These biomarkers are reflective of glycaemic control, insulin resistance and renal function.

Given that there are no effective treatments for preeclampsia, the research community has been focused on developing potential therapeutic strategies. In this review Murray et al. discussed an immunomodulatory treatment strategy that targets the CD4^+^ T cell mechanism. This mechanism-based approach associated with preeclampsia targets placental inflammation (Aneman et al., 2020), which, if ameliorated, could improve spiral artery invasion, placentation, and maternal tolerance.

### Digital Technologies for Women’s Health (2)

Digital health and remote monitoring technologies are rapidly emerging for women’s health application and could be useful particularly for high-risk pregnancies requiring close surveillance. One such application includes the management of gestational diabetes mellitus, which affects around 1 in 8 pregnant women (Hod et al., 2015). This systematic review Bertini et al. outlines the benefits of remote monitoring technologies including: improved glycaemic control, increased satisfaction and acceptability, maternal confidence, decreased gestational weight gain, knowledge of gestational diabetes mellitus, and improved medical team time management.

An article Hurst B. S. et al. in our Research Topic also reported the accuracy of a novel skin-worn sensor and its associated algorithm in determining the fertile window and absence of ovulation in 80 women. This innovative skin-sensor was compared directly to a vaginal sensor and its algorithm, showing that it could be a useful tool for women with ovarian dysfunction who are trying to conceive.

## Conclusion

In summary, this Research Topic on “*New Technologies for Women’s Health*” presents articles on different types of conditions affecting women, from challenges with conception to pregnancy complications and ovarian cancer. These articles describe breakthrough science and innovative technologies that could improve the health of women globally.
